# Association of candidate gene polymorphisms and TGF-beta/IL-10 levels with malaria in three regions of Cameroon: a case–control study

**DOI:** 10.1186/1475-2875-13-236

**Published:** 2014-06-16

**Authors:** Tobias O Apinjoh, Judith K Anchang-Kimbi, Clarisse Njua-Yafi, André N Ngwai, Regina N Mugri, Taane G Clark, Kirk A Rockett, Dominic P Kwiatkowski, Eric A Achidi

**Affiliations:** 1 Department of Biochemistry and Molecular Biology, University of Buea, Buea, Cameroon; 2 Department of Zoology and Animal Physiology, University of Buea, Buea, Cameroon; 3 Department of Animal Biology and Physiology, University of Yaounde I, Yaounde, Cameroon; 4 Department of Medical Laboratory Science, University of Buea, Buea, Cameroon; 5 London School of Hygiene and Tropical Medicine, London, UK; 6 Wellcome Trust Centre for Human Genetics, University of Oxford, Oxford, UK

**Keywords:** Single nucleotide polymorphism, Severe malaria, Uncomplicated malaria, Cytokines, Children, Ethnicity

## Abstract

**Background:**

*Plasmodium falciparum* malaria is one of the most widespread and deadliest infectious diseases in children under five years in endemic areas. The disease has been a strong force for evolutionary selection in the human genome, and uncovering the critical host genetic factors that confer resistance to the disease would provide clues to the molecular basis of protective immunity and improve vaccine development initiatives.

**Methods:**

The effect of single nucleotide polymorphisms (SNPs) and plasma transforming growth factor beta (TGF-β) and interleukin 10 (IL-10) levels on malaria pathology was investigated in a case–control study of 1862 individuals from two major ethnic groups in three regions with intense perennial *P. falciparum* transmission in Cameroon. Thirty-four malaria candidate polymorphisms, including the sickle cell trait (*HbS*), were assayed on the Sequenom iPLEX platform while plasma TGF-β and IL-10 levels were measured by sandwich ELISA.

**Results:**

The study confirms the known protective effect of *HbS* against severe malaria and also reveals a protective effect of SNPs in the nitrogen oxide synthase 2 (NOS2) gene against malaria infection, anaemia and uncomplicated malaria. Furthermore, *ADCY9* rs10775349 (additive G) and *ABO* rs8176746 AC individuals were associated with protection from hyperpyrexia and hyperparasitaemia, respectively. Meanwhile, individuals with the *EMR1* rs373533 GT, *EMR1* rs461645 CT and *RTN3* rs542998 (additive C) genotypes were more susceptible to hyperpyrexia while both females and males with the rs1050828 and rs1050829 SNPs of *G6PD,* respectively, were more vulnerable to anaemia. Plasma TGF-β levels were strongly correlated with heterozygosity for the *ADCY9* rs2230739 and *HBB* rs334 SNPs while individuals with the *ABO* rs8176746 AC genotype had lower IL-10 levels.

**Conclusion:**

Taken together, this study suggests that some rare polymorphisms in candidate genes may have important implications for the susceptibility of Cameroonians to severe malaria. Moreover using the uncomplicated malaria phenotype may permit the identification of novel pathways in the early development of the disease.

## Background

Malaria affects about one quarter of a billion people annually, with up to two-thirds of a million deaths still occurring per year, particularly in sub-Saharan African children below five years of age [1]. Why only a small proportion (1–3%) of *Plasmodium falciparum* infections progress to severe or fatal episodes [2] while others remain asymptomatic or develop an uncomplicated illness is not yet fully understood. Epidemiological data indicate that about 25% of the risk to *Plasmodium* infection in Africa is determined by human genetic factors [3]. Nevertheless, haemoglobin S, the strongest known resistance genetic factor, explains only 2% of the total variation [3], suggesting the existence of many unknown protective genes, each individually having small population effects. Single-nucleotide polymorphisms (SNPs) comprise a large part of human diversity, and their inheritance may alter susceptibility to disease [4].

Many of the malaria protective associations described to date relate to genes that affect cytokine and toll-like gene expression [5,6], red blood cell (RBC) structure or function [7]. However, there are a number of interesting candidate polymorphisms that have been associated with other infectious diseases and may be linked to malaria pathogenesis. Complement factor 6 (*C6*), for instance, shows polymorphism resulting in deficiency of the protein [8] although its role in malaria has not been proven. The susceptibility to typhoid fever is associated with a polymorphism in the cystic fibrosis transmembrane conductance regulator (*CFTR*) [9], while genetic associations of a member of the tripartite motif (*TRIM*) family with human immunodeficiency virus type 1 infection [10] have been reported. The levels of reticulon 3 (*RTN3*) is significantly increased in malaria and other infections [11] suggesting that it may be linked to the disease.

The effect of some polymorphisms on malaria pathogenesis still remains controversial. There was no association between intercellular adhesion molecule 1 gene polymorphisms and severe malaria in a West African population [12], although the SNP had earlier been reported to predispose to cerebral malaria in Kenya [13]. The *NOS2A*-954C allele has been associated with protection from severe malaria in Gabonese individuals [14,15], but studies in The Gambia and Tanzania failed to detect such a disease association [16,17]. Additionally, the *NOS2A*-1173 T allele which appears, on the basis of measurements in urine and plasma, to be associated with high NO production in children is associated with protection from malarial illness in Tanzania and from severe malarial anaemia in Kenya [18], but no protective effect against severe malaria was detected in The Gambia [17].

Several studies have demonstrated the critical role of anti-inflammatory cytokines in the immuno-pathogenesis of severe malaria anaemia (SMA) and cerebral malaria (CM). *Plasmodium chabaudi chabaudi* infected mice deficient in interleukin-10 (IL-10) show higher mortality than their normal littermates [19], suggesting a protective role for this cytokine. Furthermore, IL-10 seems to induce and maintain immunity to *P. falciparum* in naturally exposed populations [20]. Importantly, the down regulation of TNF-α production and consequent resistance to severe malaria, has been linked to the ability to produce the immuno-regulatory cytokine, Transforming growth factor (TGF)-β [21]. Low levels of IL-10 and TGF-ß have been associated with severe malaria [22,23]. Functional polymorphisms in the promoter and/or coding region(s) of cytokine genes may, therefore, be crucial in the development and clinical course of malaria [5]. Indeed, polymorphisms in genes encoding IL-10 [6,24] have been associated with susceptibility to malaria, although their functional role in severe malaria still remains open to question. How the levels of these cytokines vary in different haemoglobin β-globin and other malaria candidate SNP genotypes, therefore, warrants further investigation.

Case–control studies have been vital in detecting several genes associated with malaria or severe malaria [6,25-27]. However, some reports have been contradictory, due partly, to the analysis of small sample numbers, and hence limited statistical power. Furthermore, differences in transmission intensities or other epidemiologic characteristics at the different sites and ethnicities may affect the detection of modest effects of susceptibility or resistance genes. Some rare SNPs were investigated in a case–control study among 971 children with malaria and 891 unmatched apparently healthy control school children and blood bank donors in a bid to identify novel ones that may be linked to malaria and TGF-ß and IL-10 levels.

## Methods

### Study area

This cross-sectional study was conducted in four towns distributed in three regions of Cameroon, namely: Yaoundé in the Centre; Douala in the Littoral; and Buea and Limbe in the South West. The study sites included hospitals (Bota District Hospital - Limbe, Laquintinie Hospital - Douala, Mother and Child Hospital – Yaoundé, Regional Hospital - Limbe and Regional Hospital - Buea) and health centres (Bokova Health Centre, Mount Mary Health Centre - Buea and PMI Down Beach - Limbe). All chosen health facilities, except Mount Mary, were the main government institutions in the selected towns, also receiving patients from surrounding areas. Although malaria is endemic throughout Cameroon, the country has very different geographical and epidemiologic strata that may alter the course of the infection. In general, malaria transmission is intense and perennial in the Centre, Littoral (Coastal), and South Western regions, with peak periods corresponding to the rainy seasons [28].

The Centre region (Yaoundé) is located within the rainforest belt of central Africa [29] and has the Guinea-type equatorial climate [28]. This is characterized by fairly constant temperatures [ranging from 17°C to 30°C (mean = 23.1°C)] [30], abundant rainfall (1,500–2,000 mm), with the average relative humidity index ranging from 85% to 90%, and four distinct seasons: two rainy seasons (March – May/June, September – November) and two dry seasons (December – February, June/July – August). Maximal transmission of malaria occurs during and immediately following the two rainy seasons [28-30]. The Mother and Child Hospital is a referral hospital for children and mothers, located in the heart of the city of Yaoundé; it also attracts patients from neighbouring villages such as Simbok and Etoa that are stable, rural, farming communities with fields irrigated by water from the Mefou and Biyeme Rivers. Inhabitants of this region are of the Ewondo tribe and part of the Bantu ethnic group.

The South Western and Littoral regions have a Cameroonian-type equatorial climate characterized by fairly constant temperatures and two seasons: a short dry season (November – March) and a long rainy season (March - November) with abundant precipitation (2,000–10,000 mm) [28]. In the Mt Cameroon region of the South West, the mean annual rainfall is 2625 mm, relative humidity is constantly high (75%–80%), and the temperature varies from 18°C in August to 35°C in March [31]. Human malaria is meso-endemic during the dry season but becomes hyper-endemic in the rainy season, with incidence peaking in July–October. The prevalence of malaria parasitaemia in the low-altitude areas ranges from 30% in the dry season to 84% in the rainy season [32,33]. *Plasmodium falciparum* accounts for up to 96% of malaria infections in this area [34], with *Anopheles gambiae s.s.* the dominant vector [31].

### Study design and population

**Figure 1:**
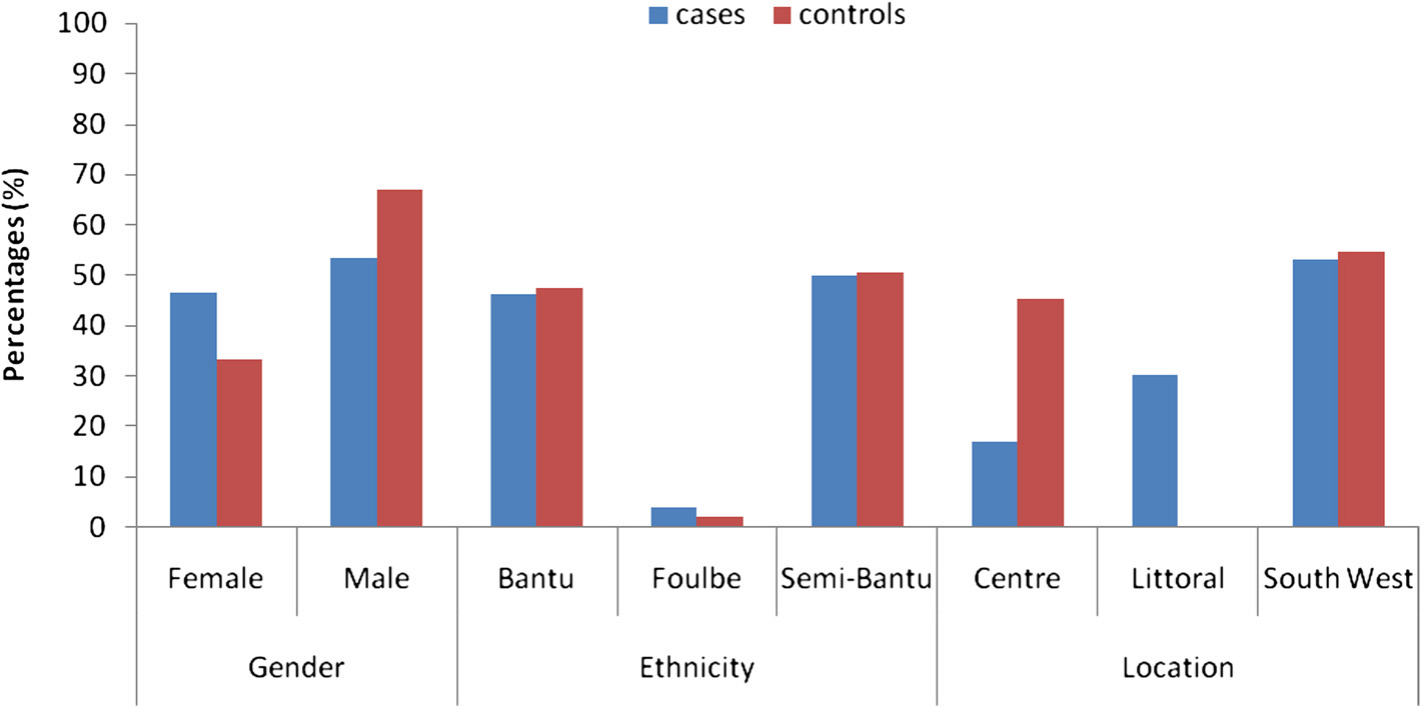
Basic demographic characteristics of the cases (n = 971) and controls (n = 891) in the study.

The study conducted between 2003/05 and 2007/08 involved malaria diagnosed children (aged 1 month – 13 years) admitted in nine health facilities in the Centre, Littoral and South West regions of Cameroon [35]. The 971 unrelated sick children sampled included severe and uncomplicated malaria cases, mainly from the Bantu and Semi-Bantu ethnic groups, recruited from the paediatric wards of the hospitals or health centres (Figure 1). In line with WHO guidelines [36], severe malaria was defined by the presence of asexual *Plasmodium* parasitaemia and at least one of the following conditions: cerebral malaria (CM) [impaired consciousness or unrousable coma (Blantyre coma score ≤ 2) and no record of recent severe head trauma, neurological disease or any other cause of coma]; severe malaria anaemia (SMA) [haemoglobin < 5 g/dL or haematocrit < 15%, no cases of severe bleeding or observed convulsions]; hyperpyrexia (axillary temperature ≥ 40°C); hyperparasitaemia (>250,000 parasites/μL); convulsions before/during admission; respiratory distress (RD) (presence of alar flaring, intercostals or subcostal chest recession, use of accessory muscles of respiration, or abnormally deep respiration) and hypoglycaemia (blood glucose <2.2 mmol/L/40 mg/dl). Participants with co-existing severe or chronic medical conditions (e.g. bacterial pneumonia, kwashiorkor) unrelated to a severe malarial infection were excluded. UM was defined as a clinical illness characterized by an axillary temperature ≥37.5°C associated with a *Plasmodium* positive blood film, haemoglobin ≥ 8 g/dL and full consciousness, in the absence of clinical signs and symptoms of severe malaria and/or evidence of vital organ dysfunction.Controls (n = 891) consisted of apparently healthy children (aged 1-14 years, afebrile and free from any obvious illness) and adults (aged 17-52 years, asymptomatic, from the community) also belonging primarily to the Bantu and Semi-Bantu ethnic groups (Figure 1). Children were recruited during malaria cross-sectional surveys from primary schools located in the South West region (Buea Metropolis) between 2004-2005 and 2007-2008. Children with parasitaemia and a temperature of 37.5°C or above were not recruited as controls. Adults were identified from a blood bank in the Centre region (Mother and Child Hospital - Yaounde) between July and August 2007. These controls were thought to approximate a random sample of the population thus, reflecting the true allele frequency. Data on the Foulbe ethnic group who constitute a significant proportion of the ethno-demographics of Cameroon were not included in the final analysis because of their small sample size in the study population.

### Ethical approvals

Ethical and administrative clearance for the study was obtained from the University of Buea Institutional Review Board and the South West Regional Delegation of Public Health respectively. Authorization to conduct the surveys in designated primary schools was obtained from the Regional Delegation of Basic Education or the Catholic Education Secretariat of the South West Region. Individuals who fulfilled the specific inclusion criteria and volunteered to participate after adequate sensitisation on the project objectives, methods and possible benefits/risks were enrolled in to the study. A health facility or school was only investigated with the approval of its Director or Head Teacher and study participants were only enrolled if they or their caregivers/guardians gave written informed consent/assent.

### Malaria parasitaemia determination

Thick and thin blood smears were prepared following standard procedures and stained with 5% Giemsa (Sigma, St. Louis, USA). The malaria parasitaemia status and density were determined under oil immersion with the 100x objective, 10x eyepiece of a binocular Olympus microscope (Olympus Optical Co., Ltd, Japan) while the *Plasmodium* species was identified on the thin blood smear. A smear was only considered negative if no malaria parasites were seen in 50 high power fields. With each positive smear, the level of parasitaemia was estimated by counting the parasites against at least 200 leucocytes and using the corresponding leucocyte count to calculate the number of parasites/μl blood [37].

### Cytokine measurement

Plasma levels of IL-10 and TGF-β were measured in cases and controls by Enzyme-Linked Immunosorbent Assay (ELISA), according to the manufacturer’s instructions (Quantikine R&D systems). Samples were obtained at the time of admission (CM/SMA), outpatient treatment (UM), or enrollment (Controls). The results were expressed in pg/ml by reference to standard curves prepared in each plate with recombinant cytokines. In all, the ELISAs, standards/samples were run in duplicates, and tested 10 non-immune Swedish & British sera were used as negative controls to check that the response was specific to malaria infection.

### Selection of SNPs and genotyping

Genomic DNA was extracted from whole blood or packed cells using the Promega Wizard (Promega Corporation, Madison, USA) or Nucleon™ BACC Genomic DNA Extraction (Gen-Probe Life Sciences, Manchester, UK) kits and quantified by the picogreen assay. The DNA samples were whole-genome amplified by Primer Extension Pre-amplification [38] before genotyping on the Sequenom IPLEX genotyping platform (Sequenom Inc., San Diego, USA) [39]. Polymorphism sequence information was downloaded from Ensembl (http://www.ensembl.org) and reformatted for the assay design process (http://www.sequenom.com). Multiplex design for the iPLEX methodology was then undertaken using the MassARRAY^®^ Assay Design v3.1 Software and the resulting multiplexes tested using a panel of CEPH and YRI HapMap DNAs. Thirty-four malaria candidate polymorphisms in various genes including *GBP7*, *DARC*, *CR1*, *C6*, *CTL4*, *NOD1*, *CD36*, *CFTR*, *ABO*, *HBB*, *HbC*, *TRIM5*, *RTN3*, *SPTB*, *ADCY9*, *ADORA2B*, *NOS2*, *EMR1*, *ICAM1*, *GNAS*, *DERL3*, *CD40LG* and *G6PD* were genotyped in 971 cases and 891 controls. The SNPs were compiled on the basis of a review of reports of associations with severe malaria or association with other infectious diseases. The haemoglobin variant S (HbS) polymorphism, known to be strongly associated with severe malaria [25,40] was also genotyped. Genotype calling was performed using the Sequenom Spectro-typer software, an automated algorithm, followed by a careful visual inspection of the genotype cluster plots to assess quality. All the assays were quality filtered (call rate > 90%) and assessed for evidence of genotypic deviation from Hardy–Weinberg equilibrium (HWE) in the controls (P < 0.001), indicative of genotyping error [41].

### Data analyses

Statistical analyses were performed using IBM SPSS Statistics 17.0 (IBM Corporation, NY, USA) and the R software package (http://www.r-project.org). Genotypic deviations from HWE were assessed using a Chi-square statistical test. Case–control association analysis of SNP alleles and genotypes was undertaken by logistic regression with self-reported ethnicity age, sex and the *HbS* polymorphism included as covariates. Adjustment for self-reported ethnicity has been found to be a robust approach to controlling the potentially confounding effects of population structure [25]. Performing multiple statistical tests can lead to an increased chance of false positives. Also, as there are multiple polymorphisms in some genes, some statistical tests will be correlated. Thus, for the genetic results, a simulation was performed by permuting the phenotypes 10000 times to establish a significance cut-off, and found a threshold of *p* ≤ 0.01 to be equivalent to a nominal false positive rate of 5%.

## Results

A total of 971 cases and 891 controls were enrolled into the study. The mean age (±standard deviation) of the cases and control children was 55.4 ± 37.9 and 87.7 ± 22.9 months respectively. The basic demographic characteristics of the study population (Figure 1) indicates a fairly gender balanced distribution in the cases [453 (46.7%) females vs. 516 (53.3%) males] that were enrolled mainly from the South West region [515 (53.3%)] and from the Semi-Bantu [416 (50.0%)] and Bantu [385 (46.3%)] ethnic groups. The most prevalent clinical phenotypes included severe malaria anaemia [n (%), 248 (21.8)], uncomplicated malaria [252 (22.1)], hyperpyrexia [116 (15)], hyperparasitaemia [58 (6.5)] and cerebral malaria [51 (5.4)].

Three polymorphisms were removed from the analysis because they were monomorphic (7:080302110, rs33950507, rs1799969) while two of the assays did not pass the quality filters (call rate > 90%, rs8176719, rs33930165), leaving 30 SNPs that could be analysed for their association with malaria phenotype (Table 1). There was no evidence of genotypic deviation from HWE in the controls (p > 0.06). Additional file 1 shows the minimum p-values from allelic/genotypic tests applied to the autosomal SNPs, and confirms that the sickle cell (HbS) polymorphism (rs334) was significantly associated with protection from malaria infection [AT *vs.* AA/TT, odds ratio (OR) = 0.29, 95%CI 0.21-0.42, p = 4.95 × 10^-13^] and from SMA [AT vs. AA/TT, OR = 0.34, 95%CI 0.13-0.93, p = 0.024] (Table 2).

**Table 1:** Minor allele frequencies and test of Hardy-Weinberg equilibrium in selected candidate SNPs

**Gene**	**Alternate SNP name**	**SNP**	**Location**	**Minor allele frequency**			**HWE**^**†**^

				**Maj/Min**	**Cases**	**Controls**	**(P value)**
					**(n = 971)**	**(n = 891)**	
HBB	HbS	rs334	Genic	A/T	0.054	0.088	0.189
GBP7		rs1803632	P	C/G	0.474	0.497	0.074
DARC		rs2814778	UTR	A/G	0.006	0.003	1.000
CR1		rs17047660	UTR	A/G	0.283	0.301	0.409
CR1		rs17047661	Genic	G/A	0.277	0.272	0.379
C6		rs1801033	Genic	A/C	0.415	0.417	0.515
CTL4		rs2242665	Genic	A/G	0.319	0.324	0.642
NOD1		rs2075820	P	G/A	0.401	0.397	0.170
CD36		rs3211938	Genic	T/G	0.144	0.148	0.409
CFTR		rs17140229	Genic	C/T	0.401	0.408	0.129
ABO		rs8176746	Genic	C/A	0.166	0.170	0.173
TRIM5		rs7935564	Genic	G/A	0.486	0.486	0.273
RTN3		rs542998	Genic	C/T	0.379	0.384	0.375
SPTB		rs229587	Genic	T/C	0.290	0.291	0.669
ADCY9		rs2230739	Genic	A/G	0.092	0.104	0.434
ADCY9		rs10775349	Genic	C/G	0.151	0.169	0.462
ADORA2B		rs2535611	Genic	T/C	0.099	0.110	0.715
NOS2		rs2297518	Genic	G/A	0.105	0.139	0.552
NOS2	NOS2-954	rs1800482	UTR	G/C	0.082	0.080	0.812
NOS2		rs9282799	UTR	C/T	0.040	0.036	0.076
NOS2	NOS2-1659	rs8078340	UTR	C/T	0.270	0.280	0.200
EMR1		rs373533	Genic	G/T	0.444	0.463	0.942
EMR1		rs461645	Genic	C/T	0.446	0.468	1.000
ICAM1	ICAM-1codon469	rs5498	Genic	A/G	0.119	0.141	0.238
GNAS	GNAS_8386	rs8386	Genic	C/T	0.159	0.154	0.224
DERL3		rs1128127	Genic	A/G	0.464	0.457	0.481
CD40LG_(F)_		rs3092945	P	T/C	0.295	0.269	0.359
CD40LG_(M)_		rs3092945	P	T/C	0.295	0.257	< 0.001
CD40LG_(F)_		rs1126535	Genic	T/C	0.188	0.161	0.190
CD40LG_(M)_		rs1126535	Genic	T/C	0.159	0.216	< 0.001
G6PD_(F)_		rs1050829	P	T/C	0.360	0.333	0.342
G6PD_(M)_		rs1050829	P	T/C	0.360	0.341	< 0.001
G6PD_(F)_		rs1050828	P	C/T	0.031	0.089	0.711
G6PD_(M)_		rs1050828	P	C/T	0.120	0.106	< 0.001

*UTR* 3’untranslated region, *P* promoter, Maj/Min = Major/Minor allele**,** M = male, F = Female ^**†**^One degree of freedom *χ*2 test of HWE applied to the 891 controls.

**Table 2:** Genotype associations between selected SNP and syndromes of malaria

**Phenotype**	**Gene**	**SNPs**	**Model**	**Genotypes**	**OR**	**95%CI**		**P value**^**‡**^
Anemia	hHbS	rs334	Heterozygous	AT vs. AA/TT	0.50	0.31	0.80	0.004
	GBP7	rs1803632	Heterozygous	CG vs. GG/CC	1.42	1.05	1.91	0.023
	CFTR	rs17140229	Additive	TT vs. CT vs. CC	0.78	0.62	0.97	0.026
	NOS2	rs2297518	Heterozygous	AG vs. AA/GG	0.49	0.35	0.70	6.32 × 10^-5^
	GNAS	rs8386	Heterozygous	CT vs. TT/CC	0.67	0.47	0.95	0.024
	G6PD_(F)_	rs1050828	Dominant	CT/TT vs. CC	2.47	1.34	4.56	0.003
	G6PD_(M)_	rs1050828	Recessive	CC vs. CT/TT	3.01	1.26	7.18	0.009
	G6PD_(F)_	rs1050829	Dominant	CT/TT vs. CC	1.88	1.20	2.93	0.005
CM	CD40LG_(F)_	rs3092945	Additive	TT vs. CT vs. CC	4.03	1.19	13.79	0.021
	CD40LG_(M)_	rs3092945	Additive	TT vs. CT vs. CC	4.03	1.18	13.79	0.021
Hyperparasitaemia	GBP7	rs1803632	Dominant	CG/CC vs. GG	0.53	0.29	0.96	0.041
	ABO	rs8176746	Heterozygous	AC vs. AA/CC	0.41	0.18	0.93	0.019
	NOS2	rs8078340	Heterozygous	CT vs. CC/TT	1.80	1.01	3.21	0.046
	DERL3	rs1128127	Recessive	AA vs. GA/GG	0.45	0.21	0.98	0.029
Hyperpyrexia	GBP7	rs1803632	Additive	GG vs. CG vs. CC	0.73	0.55	0.99	0.042
	CD36	rs3211938	Recessive	GG vs. GT/TT	2.80	1.20	6.51	0.027
	ABO	rs8176746	Heterozygous	AC vs. AA/CC	0.61	0.37	1.01	0.045
	RTN3	rs542998	Additive	TT vs. CT vs. CC	1.47	1.09	1.99	0.011
	ADCY9	rs10775349	Additive	CC vs. CG vs. GG	0.02	0.01	0.04	1.35 × 10^-87^
	EMR1	rs373533	Heterozygous	GT vs. GG/TT	1.86	1.22	2.84	0.003
	EMR1	rs461645	Heterozygous	CT vs. CC/TT	1.75	1.15	2.67	0.008
	CD40LG_(F)_	rs3092945	Dominant	CT/CC vs. TT	1.96	1.01	3.83	0.044
	G6PD_(M)_	rs1050829	Recessive	CC vs. CT/TT	1.84	1.06	3.20	0.033
SMA	hHbS	rs334	Heterozygous	AT vs. TT/AA	0.34	0.13	0.93	0.024
	ADCY9	rs2230739	Heterozygous	AG vs. GG/AA	0.48	0.23	1.02	0.050
	EMR1	rs461645	Recessive	TT vs. CT/CC	2.05	1.12	3.75	0.020
UM	NOS2	rs2297518	Heterozygous	GA vs. GG/AA	0.50	0.29	0.84	0.007
	NOS2	rs1800482	Recessive	GG vs. GC/CC	7.94	1.06	59.50	0.043
	EMR1	rs461645	Additive	CC vs. CT vs. TT	1.42	1.04	1.93	0.025

^**‡**^Additive, dominant, recessive and heterozygous advantage genotypic tests were performed, adjusted for age, sex, ethnicity and HbS, but only the most statistically significant result is presented. UM = uncomplicated malaria.

The presence of the rs2297518 SNP in the gene encoding *NOS2* was associated with protection from malaria infection (GG *vs.* AG *vs.* AA, OR = 0.52, 95%CI 0.36-0.75, p = 0.0005) (Additional file 1). In addition, individuals with the AG genotype of this SNP were protected from anaemia (OR = 0.49, 95%CI 0.35-0.70, p = 6.32 × 10^-5^) and UM (OR = 0.50, 95%CI 0.29-0.84, p = 0.007) (Table 2). Furthermore, individuals with the rs10775349 SNP of *ADCY9* were protected from hyperpyrexia (CC *vs.* CG *vs.* GG, OR = 0.02, 95%CI 0.01-0.04, p = 1.35 × 10^-87^) while heterozygosity for the rs8176746 SNP in the *ABO* locus was associated with protection against hyperparasitaemia (AC *vs.* AA/CC, OR = 0.41, 95%CI 0.18-0.93, p = 0.019) (Table 2 and Additional file 2). Nevertheless, a number of marginal protective genotype associations were also observed between specific gene mutations and anaemia (*CFTR*, *GNAS*), hyperparasitaemia (*DERL3*, *GBP7*), hyperpyrexia (*GBP7*, *ABO*) and SMA (*HbS*, *ADCY9*) (Table 2).

Male hemizygotes of *G6PD* rs1050828 (OR = 3.01, 95%CI 1.26-7.18, p = 0.009) and females with rs1050828 (CT/TT *vs.* CC, OR = 2.47, 95%CI 1.34-4.56, p = 0.003) and rs1050829 (CT/TT *vs.* CC, OR = 1.88, 95%CI 1.20-2.93, p = 0.005) SNPs of *G6PD* were more susceptible to anaemia (Table 2). Heterozygous GT and CT individuals for the rs373533 (GT *vs.* GG/TT, OR = 1.86, 95%CI 1.22-2.84, p = 0.003) and rs461645 (CT *vs.* CC/TT, OR = 1.75, 95%CI 1.15-2.67, p = 0.008) SNPs respectively in the *EMR1* gene were more likely to develop hyperpyrexia. Furthermore, individuals with the *RTN3* rs542998 SNP (additive C, OR = 1.47, 95%CI 1.09-1.99, p = 0.011) were also independently associated with susceptibility to hyperpyrexia (Table 2, Additional file 2). Even so, a number of marginal susceptibility genotype associations were also observed between specific gene mutations and anaemia (*GBP7*), CM (*CD40LG*), hyperparasitaemia (*NOS2*), hyperpyrexia (*CD36*, *CD40LG, G6PD*), SMA (*EMR1*) and UM (*NOS2*, *EMR1*) (Table 2).

Additional file 2 shows that some polymorphisms were associated with severe malaria only in individuals from the Semi-Bantu ethnic group. The sickle cell trait was significantly associated with protection from SMA [AT *vs.* AA/TT, OR = 0.33, 95%CI 0.16-0.72, p = 0.003] while individuals with the *EMR1* rs461645 TT and *NOS2* rs8078340 CT genotypes were more susceptible to SMA (OR = 2.79, 95%CI 1.30-6.02, p = 0.007) and hyperparasitaemia (OR = 2.91, 95%CI 1.24-6.83, p = 0.012) respectively. Conversely, the association between *G6PD* rs1050828 with anaemia in females (OR = 4.77, 95%CI 1.80-14.21, p = 0.003) was only observed in the Bantu ethnic group.

**Figure 2:**
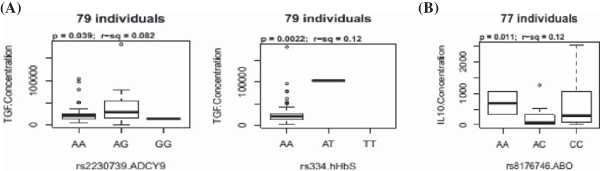
**Association between human cytokine genetic polymorphisms and plasma cytokine levels (pg/ml). A** = TGF-β and **B** = IL-10.

Plasma TGF-β levels were strongly correlated with heterozygosity for the *ADCY9* rs2230739 (p = 0.039) and *HBB* rs334 (p = 0.002) SNPs (Figure 2). Individuals with the AG genotype of the *ADCY9* gene had significantly higher levels of TGF-β compared to their AA and GG counterparts. Similarly, levels of the cytokine were higher in the AT heterozygotes of rs334 compared to their AA counterparts. Plasma IL-10 levels were also correlated with heterozygosity for the *ABO* rs8176746 (p = 0.011), with AC genotypes, however, having lower levels of the cytokine compared to their AA and CC counterparts. Nevertheless, no other SNPs were associated with levels of these cytokines.

## Discussion

The clinical outcome of human malaria infection is highly variable and heterogeneous, depending on many factors including age, transmission intensity, parasite strain virulence, co-infections with other pathogens, socio-economic status as well as the genetic background of the human host [40]. The human genome has, therefore, evolved under selective pressure exerted by pathogens, with human genetics thought to explain 25% of the inter-individual variation in susceptibility to and manifestation of malaria attributable to various host factors [3]. This natural selection process is epitomized in the historical exposure to *falciparum* malaria and its association with the β-globin SNP underlying the haemoglobin S sickle-cell trait [42]. This mutant allele, the strongest known resistance genetic factor, can reach a frequency of 25% in some populations in Sub-Saharan Africa [40] yet explains only 2% of the total variation [3]. Many unknown protective genes thus exist, each presumably individually having small population effects.

Genetic association studies utilising polymorphic markers in candidate genes have been successful in identifying a number of genes that are associated with susceptibility to malaria, or severe malaria. A large case–control study of severe malaria in children across different regions and major ethnic groups in Cameroon has the potential to confirm previously reported malaria/genetic associations or permit the identification of new ones following the assessment of association of SNPs of human genes with clinical phenotypes. This study focused on genes whose association with malaria remain controversial as well as those reportedly associated with susceptibility to other infectious diseases. This study suggests that the protective effect of the sickle cell trait may be linked to the raised level of TGF-ß and provides additional support for a role of *ABO* and *RTN3* against severe malaria. It further provides the first evidence that polymorphisms in *NOS2* and *EMR1* may be associated with mild malaria, *G6PD* with anaemia while SNPs in *ADCY9* and *EMR1* may be linked with severe malaria in the Cameroonian population. Data reported here provide additional understanding on human candidate genes that may contribute to malaria susceptibility, help define the basis of individual and population variation in susceptibility to the disease and thus eventually facilitate efforts to develop vaccines and treatments to fight this infectious disease. However, it is possible that an apparent association with malaria can arise from linkage disequilibrium between the typed SNP and a primarily associated polymorphism. Constructing a detailed map of polymorphisms around these candidate genes and describing a profile of linkage disequilibrium among them will be helpful to identify the causal variant(s). In addition, an appropriately designed family-based study may provide useful linkage information, have more power to detect association and allow haplotypes to be constructed with more confidence.

Although the CCTTT pentanucleotide microsatellite repeat in iNOS promoter is thought to play a key role in the pathogenesis of severe malaria [43], the protective effects for *NOS2* SNP is quite interesting since, no previous associations between rs2297518 and malaria have been reported. The SNP has, however, been linked with non-Hodgkin lymphoma [44] and inflammatory bowel disease [45]. It is possible that the SNP may increase the binding affinity to the mutant relative to the wild-type sequence thereby increasing the basal NOS activity and subsequent parasite killing by NO [15]. Parasites are thought to invade the liver, provoking a cytokine (e.g. IFN-γ) response that will induce *NOS2* to produce NO. The constitutively high NOS activity in children with the rs2297518 A allele will provide an advantage in resisting hepatic parasite stages without the need for additional stimuli. Nitric oxide is also thought to be effective in reducing blood-stage parasite density by antibody-dependent cellular inhibition [15] triggered by the ingestion of opsonised merozoites by activated monocytes. The latter may then release toxic products and NO acting on maturing intracellular and cytoadherent parasites to limit parasitaemia density.

Adenylate cyclase (AC) type 9 gene (*ADCY9*) is an interesting candidate gene since it is critical in neuronal signalling [46] and thus may be relevant in the pathogenesis of cerebral malaria. However, Toyota and colleagues reported only a weak association of *ADCY9* gene variation with mood disorders. Although the gene has polymorphisms with large allele frequency differences (*F*_*ST*_) between HapMap YRI and CEPH, the reported rs10775349 association with hyperpyrexia is very interesting. Nevertheless, further work is needed to strengthen these findings.

The observed association between Epidermal growth factor-like module containing, mucin-like, hormone receptor-like (EMR)1 polymorphisms and hyperpyrexia, severe malaria anaemia and uncomplicated malaria is quite interesting. *EMR1* is a macrophage marker and also a transmembrane glycoprotein present in peripheral blood mononuclear cells and presumably involved in cell-cell interactions and activation of consecutive messenger cascades [47]. Gene expression of *EMR1* is increased in lipoatrophic subcutaneous abdominal adipose tissue of HIV patients with HAART-associated lipodystrophy compared to those without [48] but no genetic associations with malaria have previously been reported. There is a need to explore further the possible role of this gene in malaria pathogenesis.

Polymorphisms in the ABO blood group were associated with hyperparasitaemia and hyperpyrexia (albert marginal) but not with the other more prevalent phenotypes. Previous studies have shown that cerebral malaria cases are less likely to be of blood group O [49,50] but more likely to be of group AB in Sri Lanka [51] and type A and B in India [50] while reports from Gabon indicated reduced risk of severe malaria of blood group A individuals [52]. How, the heterozygotes remain refractory to extremely high parasitaemia and temperature levels is unclear. Further studies are required to elucidate the functional relevance of *ABO* variants on cytokines level.

Functional studies have demonstrated that a host erythrocyte G protein signal pathway may be a critical component in parasite invasion and that erythrocyte G-alpha-s protein and the malaria parasite interact at a cellular level [53]. It however remains unclear whether this interaction would impact on disease progression. The protective effect of rs8386 with anaemia observed in this study is in line with recent reports of an association between G-alpha-s gene (*GNAS*) polymorphisms with severe malaria [54], although the SNP was only significant in multi-locus associations.

Reticulon is thought to be involved in malaria pathogenesis since its gene expression significantly increases in malaria and other infections [11] while chloroquine affects its expression [55]. However, the association of rs542998 with hyperpyrexia is quite interesting since the SNP has recently been associated with malaria in a Tanzanian population [27]. Further studies are required to elucidate the role of this polymorphism with this and other syndromes of malaria.

Cytokine polymorphisms have previously been linked with their differential production and expression in malaria [5,6], with variation in promoter sequences thought to alter specific transcription factor recognition sites, transcriptional activation and cytokine production [5]. However, the association between HbS rs334 and *ADCY9* rs2230739 with plasma levels of transforming growth factor-beta as well as *ABO* rs8176746 with interleukin-10 is quite interesting since resistance to severe malaria has been linked to the ability to produce these immuno-regulatory cytokines [21,23]. How rs334 and rs8176746 affect TGF-β and IL-10 expression respectively is unclear. Nonetheless, *ADCY9* rs2230739 may act to upregulate TGFβ transcription, with the heterozygotes providing some selective advantage since the raised TGF-β levels will down-regulate proinflammatory cytokines, such as TNF, and protect against severe malaria [22,23]. This, to our knowledge is the first report of such associations and thus needs to be explored further in different settings and with larger sample numbers. It should be noted that individual differences in the levels of the TGF-β measured at a specific moment may not only result from host genetic factors predisposing to high or low production, but also for a great part from the physiological condition at that time, as well as from global immunity.

## Conclusions

This study suggests that the protective effect of the sickle cell trait may be linked to the raised level of Transforming Growth Factor beta and provides additional support for a role of *ABO* and *RTN3* against severe malaria. It also provides the first evidence that polymorphisms in *NOS2* and *EMR1* may be associated with mild malaria, *G6PD* with anaemia while SNPs in *ADCY9* and *EMR1* may be linked with severe malaria in the Cameroonian population. Polymorphisms in human genes have important implications for the outcome of paediatric malaria in Cameroon. Moreover using mild malaria clinical phenotypes may permit the identification of novel pathways in the early development of disease.

## Competing interests

The author(s) declare that they have no competing interests; that they had financial support from MalariaGEN, EVIMalaR and CANTAM for the submitted work; no financial relationships with any organizations that might have an interest in the submitted work; no other relationships or activities that could appear to have influenced the submitted work.

## Authors’ contributions

TOA, KAR, EAA, DPK conceived and designed the experiments. TOA, AJK, NC, RNM, ANN performed the experiments. TOA, TGC, MalariaGEN analysed the data. DPK, EAA, MalariaGEN, TGC contributed reagents, materials, analysis tools. TOA, AJK, NC, KAR, EAA wrote the paper. All authors read and approved the final version of the paper.

## Supplementary Material

Additional file 1 **Allelic and genotype associations between selected SNPs and malaria.** Genotypes were tested for Additive, dominant, recessive and heterozygous advantage and then adjusted for age, sex, ethnicity and HbS, but only the most statistically significant result is presented. Click here for file

Additional file 2 **Genotype associations between selected SNP and syndromes of malaria in the two major ethnic groups.** Genotypes were tested for Additive, dominant, recessive and heterozygous advantage and then adjusted for age, sex and HbS, but only the most statistically significant result is presented. Click here for file
