# Supplementary material for: Association of candidate gene polymorphisms and TGF-beta/IL-10 levels with malaria in three regions of Cameroon: a case–control study
Source: Malar J. 2014 Jun 16;13:236. doi: 10.1186/1475-2875-13-236 (PMC4077225; doi:10.1186/1475-2875-13-236)
Supplement: Additional file 1 — Allelic and genotype associations between selected SNPs and malaria. Genotypes were tested for Additive, dominant, recessive and heterozygous advantage and then adjusted for age, sex, ethnicity and HbS, but only the most statistically significant result is presented. [file 1475-2875-13-236-S1.doc]

*Additional file 1. Allelic and genotype associations between selected SNPs and malaria*

| Gene | SNPs | Allele-based tests | | | | | Genotype-based tests | | | | | | | | |
| --- | --- | --- | --- | --- | --- | --- | --- | --- | --- | --- | --- | --- | --- | --- | --- |
|  |  | Alleles | OR | 95% CI | | P value | Model | Genotypes | OR | 95% CI | | P value (Unadjusted) | OR | 95% CI | P value‡ (Adjusted) |
| HBB | rs334 | T vs. A | 0.59 | 0.45 | 0.77 | **8.77 x 10^-5^** | Heterozygous | AT vs. AA/TT | 0.30 | 0.21 | 0.43 | 1.55 x 10^-12^ | 0.34 | 0.20-0.58 | **3.08 x 10^-5^** |
| GBP7 | rs1803632 | C vs. G | 0.89 | 0.78 | 1.02 | 0.09 | Recessive | CC vs. CG/GG | 0.77 | 0.61 | 0.98 | 0.032 | 1.36 | 0.99-1.87 | 0.057 |
| DARC | rs2814778 | A vs. G | 1.81 | 0.62 | 5.32 | 0.28 | Additive | GG vs. GA vs. AA | 0.48 | 0.15 | 1.56 | 0.20 | 0.66 | 0.11-3.93 | 0.65 |
| CR1 | rs17047660 | G vs. A | 0.92 | 0.80 | 1.07 | 0.28 | Additive | AA vs. AG vs. GG | 0.93 | 0.79 | 1.08 | 0.34 | 0.85 | 0.67-1.09 | 0.20 |
| CR1 | rs17047661 | A vs. G | 1.03 | 0.88 | 1.20 | 0.72 | Dominant | GA/AA vs. GG | 0.96 | 0.65 | 1.40 | 0.82 | 0.68 | 0.36-1.31 | 0.25 |
| C6 | rs1801033 | C vs. A | 0.99 | 0.86 | 1.13 | 0.89 | Heterozygous | AC vs. CC/AA | 1.07 | 0.88 | 1.31 | 0.50 | 1.15 | 0.91-1.45 | 0.25 |
| CTL4 | rs2242665 | G vs. A | 0.98 | 0.85 | 1.13 | 0.78 | Dominant | AG/GG vs. AA | 1.10 | 0.80 | 1.51 | 0.57 | 0.8 | 0.46-1.37 | 0.41 |
| NOD1 | rs2075820 | A vs. G | 1.02 | 0.89 | 1.17 | 0.81 | Heterozygous | AG vs. AA/GG | 1.14 | 0.93 | 1.39 | 0.20 | 1.12 | 0.80-1.56 | 0.51 |
| CD36 | rs3211938 | G vs. T | 0.97 | 0.80 | 1.17 | 0.73 | Heterozygous | GT vs. GG/TT | 0.91 | 0.72 | 1.15 | 0.44 | 0.86 | 0.63-1.16 | 0.32 |
| CFTR | rs17140229 | C vs. T | 0.97 | 0.84 | 1.11 | 0.62 | Additive | TT vs. CT vs. CC | 0.95 | 0.82 | 1.10 | 0.49 | 0.88 | 0.69-1.11 | 0.28 |
| ABO | rs8176746 | A vs. C | 0.98 | 0.82 | 1.17 | 0.84 | Additive | CC vs. AC vs. AA | 0.94 | 0.78 | 1.13 | 0.53 | 0.82 | 0.61-1.10 | 0.17 |
| TRIM5 | rs7935564 | A vs. G | 1.00 | 0.88 | 1.15 | 0.98 | Heterozygous | AG vs. AA/GG | 1.18 | 0.97 | 1.44 | 1.18 | 1.16 | 0.85-1.60 | 0.35 |
| RTN3 | rs542998 | C vs. T | 0.98 | 0.85 | 1.12 | 0.74 | Heterozygous | CT vs. CC/TT | 0.87 | 0.72 | 1.07 | 0.19 | 1.27 | 0.79-2.03 | 0.32 |
| SPTB | rs229587 | C vs. T | 0.99 | 0.86 | 1.15 | 0.93 | Heterozygous | CT vs. CC/TT | 1.02 | 0.83 | 1.25 | 0.86 | 0.99 | 0.72-1.36 | 0.93 |
| ADCY9 | rs2230739 | G vs. A | 0.86 | 0.69 | 1.08 | 0.20 | Dominant | GA/GG vs. AA | 0.81 | 0.63 | 1.05 | 0.11 | 0.62 | 0.41-0.94 | 0.025 |
| ADCY9 | rs10775349 | G vs. C | 0.88 | 0.73 | 1.05 | 0.16 | Dominant | GC/GG vs. CC | 1.57 | 0.74 | 3.30 | 0.23 | 1.12 | 0.78-1.59 | 0.54 |
| ADORA2B | rs2535611 | C vs. T | 0.89 | 0.71 | 1.10 | 0.28 | Recessive | CC vs. CT/TT | 1.23 | 0.96 | 1.58 | 0.10 | 1.28 | 0.90-1.84 | 0.17 |
| NOS2 | rs2297518 | A vs. G | 0.73 | 0.60 | 0.90 | **0.003** | Additive | GG vs. AG vs. AA | 0.71 | 0.57 | 0.89 | 0.002 | 0.52 | 0.36-0.75 | **0.0005** |
| NOS2 | rs1800482 | C vs. G | 1.02 | 0.80 | 1.30 | 0.87 | Recessive | GG vs. GC/CC | 1.15 | 0.38 | 3.46 | 0.81 | 3.68 | 0.51-26.46 | 0.18 |
| NOS2 | rs9282799 | T vs. C | 1.10 | 0.78 | 1.57 | 0.58 | Additive | CC vs. CT vs. TT | 1.12 | 0.78 | 1.61 | 0.55 | 0.91 | 0.51-1.60 | 0.74 |
| NOS2 | rs8078340 | T vs. C | 0.95 | 0.82 | 1.11 | 0.54 | Dominant | CT/TT vs. CC | 0.90 | 0.74 | 1.10 | 0.30 | 0.9 | 0.19-27.62 | 0.53 |
| EMR1 | rs373533 | T vs. G | 0.93 | 0.81 | 1.06 | 0.28 | Additive | GG vs. GT vs. TT | 0.92 | 0.80 | 1.07 | 0.28 | 0.87 | 0.69-1.08 | 0.21 |
| EMR1 | rs461645 | T vs. C | 0.91 | 0.80 | 1.05 | 0.21 | Additive | CC vs. CT vs. TT | 1.09 | 0.95 | 1.26 | 0.21 | 1.35 | 0.95-1.93 | 0.09 |
| ICAM1 | rs5498 | G vs. A | 0.83 | 0.68 | 1.01 | 0.06 | Additive | AA vs. GA vs. GG | 0.84 | 0.68 | 1.04 | 0.11 | 0.72 | 0.49-1.04 | 0.08 |
| GNAS | rs8386 | T vs. C | 1.03 | 0.86 | 1.24 | 0.75 | Recessive | TT vs. CT/CC | 1.17 | 0.66 | 2.08 | 0.60 | 1.4 | 0.56-3.52 | 0.47 |
| DERL3 | rs1128127 | G vs. A | 1.03 | 0.90 | 1.18 | 0.68 | Heterozygous | GA vs. GG/AA | 1.24 | 1.01 | 1.51 | 0.038 | 1.04 | 0.83-1.31 | 0.71 |
| CD40LG _(F)_ | rs3092945 | C vs. T | 1.08 | 0.85 | 1.37 | 0.55 | Heterozygous | CT vs. CC/TT | 1.09 | 0.78 | 1.52 | 0.611 | 1.36 | 0.82-2.24 | 0.233 |
| CD40LG _(M)_ | rs3092945 | C vs. T | 1.21 | 0.99 | 1.47 | 0.06 | Heterozygous | CT vs. CC/TT | 2.72 | 0.49 | 14.92 | 0.234 | 1.46 | 0.05-42.41 | 0.822 |
| CD40LG _(F)_ | rs1126535 | C vs. T | 1.21 | 0.91 | 1.61 | 0.19 | Additive | CC vs. CT vs. TT | 1.38 | 1.00 | 1.87 | 0.046 | 1.16 | 0.72-1.85 | 0.544 |
| CD40LG _(M)_ | rs1126535 | C vs. T | 0.68 | 0.55 | 0.86 | **0.001** | Dominant | CT/TT vs. CC | 0.76 | 0.55 | 1.06 | 0.103 | 1.06 | 0.78-1.44 | 0.696 |
| G6PD _(F)_ | rs1050828 | C vs. T | - | - | - | - | Dominant | CT/TT vs. CC | 1.23 | 0.81 | 1.87 | 0.333 | 1.58 | 0.84-2.98 | 0.154 |
| G6PD _(M)_ | rs1050828 | C vs. T | - | - | - | - | Dominant | CT/TT vs. CC | 1.27 | 0.85 | 1.90 | 0.243 | 1.85 | 0.81-4.25 | 0.138 |
| G6PD _(F)_ | rs1050829 | C vs. T | 1.21 | 0.96 | 1.51 | 0.11 | Dominant | CT/TT vs. CC | 1.36 | 0.98 | 1.89 | 0.063 | 1.29 | 0.79-2.10 | 0.306 |
| G6PD _(M)_ | rs1050829 | C vs. T | 1.09 | 0.90 | 1.31 | 0.38 | Dominant | CT/TT vs. CC | 1.08 | 0.82 | 1.42 | 0.572 | 1.11 | 0.69-1.79 | 0.677 |

**^‡^** Additive, dominant, recessive and heterozygous advantage genotypic tests were performed, adjusted for age, sex, ethnicity and HbS, but only the most statistically significant result is presented
