# Supplementary material for: Association of candidate gene polymorphisms and TGF-beta/IL-10 levels with malaria in three regions of Cameroon: a case–control study
Source: Malar J. 2014 Jun 16;13:236. doi: 10.1186/1475-2875-13-236 (PMC4077225; doi:10.1186/1475-2875-13-236)
Supplement: Additional file 2 — Genotype associations between selected SNP and syndromes of malaria in the two major ethnic groups. Genotypes were tested for Additive, dominant, recessive and heterozygous advantage and then adjusted for age, sex and HbS, but only the most statistically significant result is presented. [file 1475-2875-13-236-S2.doc]

**Additional file 2. Genotype associations between selected SNP and syndromes of malaria in the two major ethnic groups**

| **Phenotype** | **Gene** | **SNPs** | **Bantu** | | | | | **Semi-Bantu** | | | | |  |
| --- | --- | --- | --- | --- | --- | --- | --- | --- | --- | --- | --- | --- | --- |
|  |  |  | **Model** | **Genotypes** | **OR** | **95% CI** | **P value^‡^** | **Model** | **Genotypes** | **OR** | **95% CI** | **P value^‡^** |  |
| Anemia | hHbS | rs334 | Heterozygous | AT vs. AA/TT | 0.57 | 0.26 - 1.26 | 0.162 | Heterozygous | AT vs. AA/TT | 0.45 | 0.24 - 0.83 | **0.009** |  |
|  | GBP7 | rs1803632 | Recessive | CC vs. CG/GG | 0.55 | 0.31 - 0.96 | 0.036 | Recessive | CC vs. CG/GG | 0.88 | 0.55 - 1.42 | 0.598 |  |
|  | CFTR | rs17140229 | Additive | TT vs. CT vs. CC | 0.80 | 0.55 - 1.16 | 0.23 | Additive | TT vs. CT vs. CC | 0.77 | 0.58 - 1.03 | 0.077 |  |
|  | NOS2 | rs2297518 | Heterozygous | AG vs. AA/GG | 0.45 | 0.26 - 0.78 | **0.005** | Heterozygous | AG vs. AA/GG | 0.53 | 0.33 - 0.85 | **0.008** |  |
|  | GNAS | rs8386 | Heterozygous | CT vs. TT/CC | 0.60 | 0.34 - 1.07 | 0.086 | Heterozygous | CT vs. TT/CC | 0.65 | 0.41 - 1.01 | 0.057 |  |
|  | G6PD _(F)_ | rs1050828 | Dominant | CT/TT vs. CC | 4.77 | 1.60-14.21 | **0.003** | Dominant | CT/TT vs. CC | 1.68 | 0.78-3.58 | 0.178 |  |
|  | G6PD _(F)_ | rs1050829 | Dominant | CT/TT vs. CC | 1.97 | 0.95-4.07 | 0.068 | Additive | CC vs. CT vs. TT | 1.57 | 1.06-2.34 | 0.023 |  |
|  | G6PD _(M)_ | rs1050828 | Recessive | CC vs. CT/TT |  |  |  | Recessive | CC vs. CT/TT | 3.13 | 1.03-9.50 | 0.037 |  |
| CM | CD40LG _(F)_ | rs3092945 | Additive | TT vs. CT vs. CC | 0.24 | 0.04 - 1.45 | 0.056 | Additive | TT vs. CT vs. CC | 3.56 | 0.80-15.95 | 0.090 |  |
|  | CD40LG _(M)_ | rs3092945 | Additive | TT vs. CT vs. CC | 0.24 | 0.04 - 1.45 | 0.056 | Additive | TT vs. CT vs. CC | 3.56 | 0.80-15.95 | 0.090 |  |
| Hyperparasitaemia | GBP7 | rs1803632 | Dominant | CG/CC vs. GG | 0.47 | 0.19 - 1.20 | 0.125 | Dominant | CG/CC vs. GG | 0.51 | 0.22 - 1.20 | 0.131 |  |
|  | ABO | rs8176746 | Heterozygous | AC vs. AA/CC | 0.62 | 0.20 - 1.90 | 0.385 | Heterozygous | AC vs. AA/CC | 0.24 | 0.06 - 1.03 | 0.021 |  |
|  | NOS2 | rs8078340 | Heterozygous | CT vs. CC/TT | 1.38 | 0.57 - 3.34 | 0.482 | Heterozygous | CT vs. CC/TT | 2.91 | 1.24 - 6.83 | **0.012** |  |
|  | DERL3 | rs1128127 | Recessive | AA vs. GA/GG | 0.67 | 0.22 - 2.04 | 0.462 | Heterozygous | GA vs. AA/GG | 2.30 | 0.97 - 5.47 | 0.051 |  |
| Hyperpyrexia | GBP7 | rs1803632 | Additive | GG vs. CG vs. CC | 0.82 | 0.51 - 1.34 | 0.434 | Dominant | CG/CC vs. GG | 0.55 | 0.32 - 0.96 | 0.038 |  |
|  | CD36 | rs3211938 | Recessive | GG vs. GT/TT | 2.31 | 0.61 - 8.67 | 0.251 | Recessive | GG vs. GT/TT | 3.85 | 1.22 - 12.16 | 0.033 |  |
|  | ABO | rs8176746 | Heterozygous | AC vs. AA/CC | 0.61 | 0.26 - 1.45 | 0.248 | Heterozygous | AC vs. AA/CC | 0.69 | 0.36 - 1.32 | 0.247 |  |
|  | RTN3 | rs542998 | Additive | TT vs. CT vs. CC | 0.99 | 0.60 - 1.64 | 0.964 | Additive | TT vs. CT vs. CC | 1.98 | 1.32 - 2.96 | **0.0008** |  |
|  | ADCY9 | rs10775349 | Additive | CC vs. CG vs. GG | 0.02 | 0.01 - 0.05 | **1.22 x 10^-32^** | Additive | CC vs. CG vs. GG | 0.02 | 0.01 - 0.05 | **2.83 x 10^-49^** |  |
|  | EMR1 | rs373533 | Heterozygous | GT vs. GG/TT | 1.24 | 0.62 - 2.46 | 0.545 | Heterozygous | GT vs. GG/TT | 2.67 | 1.49 - 4.77 | **0.0006** |  |
|  | EMR1 | rs461645 | Heterozygous | CT vs. CC/TT | 1.22 | 0.61 - 2.43 | 0.575 | Heterozygous | CT vs. CC/TT | 2.41 | 1.36 - 4.27 | **0.002** |  |
|  | CD40LG _(F)_ | rs3092945 | Dominant | CT/CC vs. TT | 2.14 | 0.68 - 6.75 | 0.186 | Dominant | CT/CC vs. TT | 1.73 | 0.72 - 4.19 | 0.219 |  |
|  | G6PD _(M)_ | rs1050829 | Recessive | CC vs. CT/TT | 2.99 | 1.17 - 7.61 | 0.021 | Recessive | CC vs. CT/TT | 1.43 | 0.69 - 2.95 | 0.342 |  |
| SMA | hHbS | rs334 | Heterozygous | AT vs. TT/AA | 0.89 | 0.19 - 4.17 | 0.881 | Heterozygous | AT vs. TT/AA | 0.33 | 0.16 - 0.72 | **0.003** |  |
|  | ADCY9 | rs2230739 | Heterozygous | AG vs. GG/AA | 0.62 | 0.18 - 2.18 | 0.448 | Heterozygous | AG vs. GG/AA | 0.43 | 0.16 - 1.14 | 0.082 |  |
|  | EMR1 | rs461645 | Recessive | TT vs. CT/CC | 1.15 | 0.40 - 3.31 | 0.80 | Recessive | TT vs. CT/CC | 2.79 | 1.30 - 6.02 | **0.007** |  |
| UM | NOS2 | rs2297518 | Heterozygous | GA vs. GG/AA | 0.72 | 0.35 - 1.46 | 0.354 | Heterozygous | GA vs. GG/AA | 0.23 | 0.09 - 0.58 | **0.0006** |  |
|  | NOS2 | rs1800482 | Recessive | GG vs. GC/CC | 7.88 | 0.53 - 116.31 | 0.116 | Recessive | GG vs. GC/CC | 7.88 | 0.16 - 10.31 | 0.826 |  |
|  | EMR1 | rs461645 | Additive | CC vs. CT vs. TT | 1.21 | 0.79 - 1.87 | 0.384 | Additive | CC vs. CT vs. TT | 1.61 | 1.01 - 2.58 | 0.041 |  |

**^‡^**Additive, dominant, recessive and heterozygous advantage genotypic tests were performed, adjusted for age, sex, and HbS, but only the most statistically significant result is presented. UM =

uncomplicated malaria; CM = Cerebral Malaria; SMA = Severe Malaria Anaemia
